# Three-Dimensional Printing of a LiFePO_4_/Graphite Battery Cell via Fused Deposition Modeling

**DOI:** 10.1038/s41598-019-54518-y

**Published:** 2019-12-02

**Authors:** Alexis Maurel, Sylvie Grugeon, Benoît Fleutot, Matthieu Courty, Kalappa Prashantha, Hugues Tortajada, Michel Armand, Stéphane Panier, Loïc Dupont

**Affiliations:** 1 Laboratoire de Réactivité et de Chimie des Solides, UMR CNRS 7314, Hub de l’Énergie, Université de Picardie Jules Verne, 15 rue Baudelocque, 80039 Amiens Cedex, France; 2 Laboratoire des Technologies Innovantes, LTI-EA 3899, Université de Picardie Jules Verne, 80025 Amiens, France; 3 RS2E, Réseau français sur le stockage électrochimique de l’énergie, FR CNRS 3459, 80039 Amiens Cedex, France; 4 IMT Lille Douai, Institut Mines-Télécom, Centre d’Enseignement, de Recherche et d’Innovation (CERI): Matériaux et Procédés Innovants, 941 rue Charles Bourseul C.S.10838, 59508 Douai Cedex, France; 5 ACU-R&D Centre, Adichunchanagiri University, Bala Gangadharanatha Nagara, 571448 Karnataka, India; 6 Plateforme de Microscopie Électronique (PME) de l’Université de Picardie Jules Verne, Hub de l’Énergie, 15 rue Baudelocque, 80000 Amiens, France

**Keywords:** Mechanical engineering, Batteries, Batteries

## Abstract

Among the 3D-printing technologies, fused deposition modeling (FDM) represents a promising route to enable direct incorporation of the battery within the final 3D object. Here, the preparation and characterization of lithium iron phosphate/polylactic acid (LFP/PLA) and SiO_2_/PLA 3D-printable filaments, specifically conceived respectively as positive electrode and separator in a lithium-ion battery is reported. By means of plasticizer addition, the active material loading within the positive electrode is raised as high as possible (up to 52 wt.%) while still providing enough flexibility to the filament to be printed. A thorough analysis is performed to determine the thermal, electrical and electrochemical effect of carbon black as conductive additive in the positive electrode and the electrolyte uptake impact of ceramic additives in the separator. Considering both optimized filaments composition and using our previously reported graphite/PLA filament for the negative electrode, assembled and “printed in one-shot” complete LFP/Graphite battery cells are 3D-printed and characterized. Taking advantage of the new design capabilities conferred by 3D-printing, separator patterns and infill density are discussed with a view to enhance the liquid electrolyte impregnation and avoid short-circuits.

## Introduction

As worldwide energy consumption is expected to increase, the supply of clean and sustainable energy is one of the most imperative scientific challenge facing humanity in the 21st century. As a result, during last decades, scientists focused their research on renewable energy sources such as wind, solar or hydroelectricity to be used in substitution of fossil fuels raising serious environmental concerns^1–7^. Meanwhile, alongside the expansion of sustainable energy sources, electrical energy storage systems^8^ capable of catching the energy produced now to supply it when needed, have lately appeared as a significant interrogation. Because of their reliable performances regarding high energy density, power density and long cycle life, lithium-ion batteries were reported to be favorable applicants amongst the electrochemical systems^8–11^. Nowadays, they are used in a wide variety of applications such as cellphones, laptops, aerospace electronics, micro-electromechanical systems, electric vehicles and hybrid vehicles^12–14^. Current technology involves anode, separator and cathode films which are rolled or stacked in a planar arrangement (2D), thus enabling lithium ion diffusion in one dimension between both electrodes^15^. However, the necessity to maximize energy storage while reducing volume and weight obliges the elaboration of methodologies to quickly design, prototype, and manufacture batteries in any desired shape, as the energy storage devices of the future are envisaged to be flexible, wearable, lightweight, and customizable^15–19^.

An approach could be the use of additive manufacturing technologies (AM) which include processes that build 3D items by addition of material layer-upon-layer^20–24^. Thanks to AM, topological optimization of energy-storage devices can be imagined^19,25^, thus allowing new types of implementations. Indeed, battery components’ shapes such as electrodes, separator, solid polymer electrolyte or also current collector can be customized, thus enabling the direct incorporation of microbatteries and electronics within the final optimized design 3D object^26–30^.

On the other hand, to reach enhanced electrochemical behavior, 3D battery designs were introduced^31,32^. 3D architecture allowing two-dimensional or three-dimensional diffusion of the lithium cations were disclosed, thus increasing the electrochemical active surface present on the same footprint area, power and specific capacity^33^. This was confirmed by Ragones *et al*.^25^ who reported the modeling of interlaced electrode 3D networks and evaluated the area gain of those architectures. More recently, an outstanding work done by Trembacki *et al*.^34^ demonstrated through simulations that 3D battery designs (gyroid and Schwarz P) perform significantly better than the particle bed geometry (2D) with energy density improvements of 3.7x − 6.9x observed at the highest power density simulated (12C).

Several groups^13,35,36^ already demonstrated the preparation of 3D-electrodes separately (positive or negative). However, as a consequence of both 3D electrodes surface roughness, the mounting of the full 3D device was described as particularly challenging with a view to prevent any short-circuit. Positive and negative electrodes interpenetration concerns may be overcome by using additive manufacturing technologies (AM). Within this framework, an escalation of investigations concerning the AM technologies, applied to energy storage field were reported lately^7,19,25,37–48^. Among them, liquid deposition modeling (LDM) and fused deposition modeling (FDM) were seriously investigated recently in order to 3D-print electrochemical storage systems such as lithium-ion batteries.

LDM is a method in which an ink employed as material source for the 3D-printer is extruded from a syringe while the latter is moved across a platform controlled by computer. Thin layers of material are deposited in order to build the desired 3D object in many successive passes. Regarding this process (Table 1), pioneering work was reported by Sun *et al*.^43^ who described the first 3D-printed Li-ion microbattery. They prepared cellulose-based inks of Li_4_Ti_5_O_12_ (LTO) and LiFePO_4_ (LFP) nanoparticles suspended in an aqueous solution for the formulation of negative and positive electrodes. Those inks were 3D-printed through a syringe, dried and sintered at 600 °C for 2 h in an inert atmosphere. Discharge electrochemical measurements for half-cell of LFP led to specific capacity of 160 mAh g^−1^ of active material at current density of 170 mA g^−1^ (1 C), corresponding to areal capacity of 1.6 mAh cm^−2^ at 1.7 mA cm^−2^. In the other hand, half-cell of LTO displayed a specific capacity of 131 mAh g^−1^ of active material at current density of 175 mA g^−1^ (1 C), equals to areal capacity of 1.4 mAh cm^−2^ at 1.87 mA cm^−2^.

**Table 1:** Summary of inks characteristics prepared for LDM process reported in literature.

Inks (Solvent)	% of AM within the total composite after post-treatment	Novelty	Post treatment	References
LFP ink and LTO ink (DI water/EG/glycerol)	∼100%	Pioneering work	600 °C for 2 h in an inert atmosphere	Sun et al.^43^
LFP/GO ink and LTO/GO ink (DI water)	∼100%	Separator ink (PVDF-co-HFP with Al_2_O_3_) + GO sheets for the viscosity	600 °C for 2 h in Ar/H_2_	Fu *et al*.^37^
LMO/CB/PVDF ink (NMP)	>85.5%wt	Electric field treatment	Electric field (10 kV) for 10 minutes + drying at 120 °C for 10 minutes	Li *et al*.^49^
LFP/KB ink and LTO/KB ink (PC)	∼100%	UV curable inks: Separator ink (PC/ Al_2_O_3_/Triton X-100) + packaging ink	UV curing steps	Wei *et al*.^51^

AM, active material; CB, carbon black; EG, ethylene glycol; KB, ketjen black; LFP, LiFePO_4_; LMO, LiMn_2_O_4_; LTO, Li_4_Ti_5_O_12_; NMP, N-methyl-2-pyrrolidone; PC, propylene carbonate; PVDF-co-HFP, poly(vinylidene fluoride-co-hexafluoropropylene).

In order to obtain the required viscosity enabling 3D printing but also to improve the electrode’s electrical conductivity, Fu *et al*.^37^ used aqueous inks of LTO/graphene oxide (GO) and LFP/GO to feed the printer. Both printed electrodes were subsequently freeze-dried to eliminate water solvent and solidify the 3D structures. A sintering procedure was applied under Ar/H_2_ (600 °C for 2 h) to allow the formation of reduced graphene oxide (rGO). The finally obtained LFP/rGO and LTO/rGO half cells were electrochemically tested using a polyvinylidene fluoride-co-hexafluoropropylene (PVDF-co-HFP) with Al_2_O_3_ 3D-printed separator. At current density of 10 mA g^−1^ (C/17), specific capacities of 164 and 185 mAh g^−1^ of active material were respectively demonstrated. These electrodes were then assembled into a full battery depicting a capacity about 100 mAh g ^−1^ during ten cycles at a specific current of 50 mA g^−1^.

Li *et al*.^49^ reported the 3D-printing of a LiMn_2_O_4_/carbon black/PVDF ink (wt.% LMO/CB/PVDF 85.5/6.5/8) dispersed in N-methyl-2-pyrrolidone solvent (NMP) to fabricate electrodes. After printing, a voltage of 10 kV was applied at a distance of 1.25 cm for 10 minutes. A hot plate (at 120 °C) was finally used to remove solutions quickly. In the proposed processing, the applied electric field controls the microstructures of manganese-based electrodes, while additive manufacturing controls macro-3D structures. As compared to a conventional laminated structure (1.8 mAh.cm^−2^ at C/10), the macro-micro controlled structure showed improved performances (3.5 mAh.cm^−2^).

As exemplified hereabove^37^, 3D-printed separator loaded with various ceramic fillers were reported in literature. Furthermore, Liu *et al*.^50^ prepared a PVDF-co-HFP/boron-nitride (BN) ink using dimethyl formamide as solvent to 3D-print separator. After printing, it was dried directly at room temperature. Authors showed that BN-separator dispenses quick heat dispersion and a homogeneous thermal distribution at the interface during the Li plating process increasing electrochemical performance of the lithium-ion battery.

Finally, an important milestone was reached by Wei *et al*.^51^ who reported 3D-printing of a fully packaged lithium-ion battery. Cathode (LFP), anode (LTO), as well as UV curable packaging and separator inks for LDM were developed. Electrodes ink compositions were 30 vol% LFP with 1.25 vol% Ketjen black (KB) conductive particles and 30 vol% LTO with 1.35 vol% KB in 1 M LiTFSI/propylene carbonate with 1 wt.% polyvinylpyrrolidone (with respect to LFP or LTO) for the cathode and anode, respectively. The separator ink was a mixture of PC/Al_2_O_3_/Triton X-100 surfactant. The cathode, anode, separator, and packaging inks were housed in separate 3 mL syringes. 3D-printing was performed by using an Ar-powered fluid dispenser to pressurize the barrel up to 700 psi to control the flow rate. After UV-curing, these fully 3D printed and packaged LIBs, which were encased between two glassy carbon current collectors, deliver an aerial capacity of 4.45 mAh cm^−2^ (equivalent to 17.3 mAh cm^−3^) at a current density of 0.14 mA cm^−2^.

To summarize, while significant advantage of LDM versus FDM is the possibility to achieve high active mass loading, main drawback of the LDM is certainly the requirement of performing post-processes such as freeze-drying and sintering prior to its use^37,43^. Furthermore, ink viscosity is a key factor to consider as it restricts the height of the successively 3D-printed object. Hence, many parameters impacting this important point must be studied thoroughly such as the introduction of surfactant and/or the active material granulometry among others.

FDM technology can overcome the latter concerns, nevertheless, only thermoplastic polymers such as polylactic acid (PLA), polyethylene terephthalate glycol (PETG), acrylonitrile-butadiene-styrene (ABS), polycarbonate (PC), or also polypropylene (PP) amongst the most employed, can be used to prepare a filament suitable for an FDM 3D-printer.

FDM produces cheap complicated items with nearly no material leftover^20,23,52^. This technology consists of depositing, through a heated nozzle of which the movement (XYZ-axis) is meticulously computer-piloted, thin layers of melted thermoplastic material in order to build the desired 3D object in many successive passes. As the thermoplastic material is heated only few degrees above its melting temperature to be deposited, it instantaneously cools down and solidifies after extrusion. Recent commercial FDM 3D-printers layer thickness resolution ordinarily reaches 200 µm for the first layer and can be commonly enhanced up to 50 µm for the successive layers. For higher resolution purposes, layer thickness resolution can be improved by customizing the machine and adjusting computer program settings^53^. FDM is now employed for fast prototyping in a wide variety of purposes comprising aerospace, automotive but also medical fields.

Regarding the FDM technique focused on lithium-ion batteries (Table 2), pioneering work was reported by Foster *et al*.^44^ in 2017. They described the fabrication of a 1 mm thick 3D-printed negative electrode disc by operating a commercial graphene-based polylactic acid filament (graphene/PLA) as material source. Because of the little amount of active material (only 8 wt.% graphene compared to 92 wt.% PLA) within the filament (103 mg of active material per cm^3^ of composite), low discharge specific capacities of 15.8 mAh g^−1^ of active material (1.26 mAh g^−1^ of the total composite equivalent to 1.63 mAh cm^−3^ considering the electrode volume) were reached at current densities of 10 mA g^−1^ (C/37).

**Table 2:** Summary of filaments characteristics prepared for FDM process reported in literature. Helium pycnometer material densities were used for weight-volume conversion.

Filaments	Total composite wt%	Total composite vol%	References
**Graphene**/PLA	**8**/92	**5**/95*	Foster *et al*.^44^
**LTO**/Carbon additives/PLA **LFP**/Carbon additives/PLA	—	—	Ragones *et al*.^25^
**LTO**/Graphene/PLA	**13**/33/54*	**6**/24/70	Reyes *et al*.^19^
**LMO**/MWNT/PLA	**11**/22/67*	**4**/16/80	
**Graphite**/PLA/PEGDME500	**63**/26/11	**49**/35/16	Maurel *et al*.^42^
**Graphite**/PLA/CSP/PEGDME500	**49**/33/5/13	**37**/40/4/19	

CSP, Carbon Super P; LFP, LiFePO_4_; LMO, LiMn_2_O_4_; LTO, Li_4_Ti_5_O_12_; MWNT, multiwalled carbon nanotubes; PEGDME500, poly(ethylene glycol) dimethyl ether average Mn ~500; PLA, polylactic acid.

*Weight-volume conversion was deduced by ourselves considering the densities obtained for analogous materials via helium pycnometer.

Ragones *et al*.^25^ pursued additional investigations by reporting the development of PLA/LTO anode and PLA/LFP cathode composite filaments. Separated electrode discs were obtained as well as spiral and double-spiral shaped current-collector/electrode (graphene-PLA/LTO-PLA) networks. Independent 3D-printed electrodes were tested in half-cells setup, soaked in 1 M LiPF_6_ in 1: 1 vol% ethylene carbonate: diethylcarbonate (EC: DEC), 2% vinylene carbonate (VC) electrolyte. The cycling of the cathode PLA/LFP resulted in 60, 50 and 20 mAh g^−1^ of LFP capacities at current density of 9, 44 and 88 µA cm^−2^, respectively.

Reyes *et al*. ^19^ reported recently the 3D printing of a complete lithium-ion battery (LTO-LMO full cell) by FDM for the first time. This was possible through the formulation of PLA/LTO/conductive additives and PLA/LMO/conductive additives filaments employed to feed the FDM 3D-printer (6 vol% for LTO anode and 4 vol% for LMO cathode). Commercial filament of PLA was used as separator and infused in 1 M LiClO_4_ in 1: 1 vol% ethyl methyl carbonate: propylene carbonate (EMC: PC). Fully 3D printed assembled coin cell, as well as “one-shot” 3D printed devices and wearable electronic devices with integrated batteries were described. The volumetric capacity of the full-cell was reported to be equals to 0.25 mAh cm^−3^ while negative and positive half-cells displayed respectively 0.34 and 0.71 mAh cm^−3^ at a current density of 10 mA g^−1^.

Unfortunately, due to the printability requirements for the FDM process, active material loading was kept relatively low in those last studies thus impacting severely the electrochemical performances^19,25,44^. However, as reported in our previous work^42^, this limitation can be overcome by introducing a plasticizer. Indeed, we reported a highly loaded 3D printable graphite/PLA filament specifically conceived to be employed as negative electrode in a lithium-ion battery and to feed a conventional FDM 3D printer. Active material content (graphite) within the filament, was increased as high as possible (49.2 wt% of graphite in the total composite thus corresponding to 773 mg of active material per cm^3^) to improve the electrochemical performances while preserving enough mechanical strength to be printed thanks particularly to the addition of poly(ethylene glycol) dimethyl ether average Mn ∼ 500 acting as plasticizer. Hence, an unprecedent reversible capacity for a negative electrode disc obtained via FDM was achieved: 200 mAh g^−1^ of active material (99 mAh g^−1^ of the total composite or also to 154.6 mAh cm^−3^) at current density of 18.6 mA g^−1^ (C/20) after 6 cycles and 140 mAh g^−1^ of active material (69 mAh g^−1^ of the total composite or also 108.2 mAh cm^−3^) at current density of 37.3 mA g^−1^ (C/10).

In order to print the complete lithium-ion battery through FDM, filament formulation of the positive electrode and separator is now required. Here, this work was focused on the development and optimization of LFP-PLA and PLA-SiO_2_ composite-based 3D-printing filaments respectively. Through the formulation process, the effect of carbon black as conductive additive in the positive electrode and the impact of ceramic additives in the separator on the ionic conductivity and swelling were investigated. Furthermore, exploiting the new design/geometry capabilities arising from 3D-printing, separator patterns were considered with a view to enhance the liquid electrolyte uptake. By using both optimized filaments as well as our previously reported graphite/PLA filament for the negative electrode, assembled and “printed in one-shot” complete LFP/Graphite battery cells are 3D-printed and characterized. Assembled cell was mounted after printing separately the different components, while a “printed in one-shot” battery was obtained by pausing the machine and changing the filament between each layer. After each formulation steps (film, filament and/or 3D-printed disc), composite electrodes were characterized thoroughly by performing thermal (DSC), mechanical (tensile test), morphological (SEM), but also electrical and electrochemical analyses. Well aware of the limitations induced by nominal resolution of 3D-printer, this work serves here as proof of concept.

## Results and Discussions

**Figure 1:**
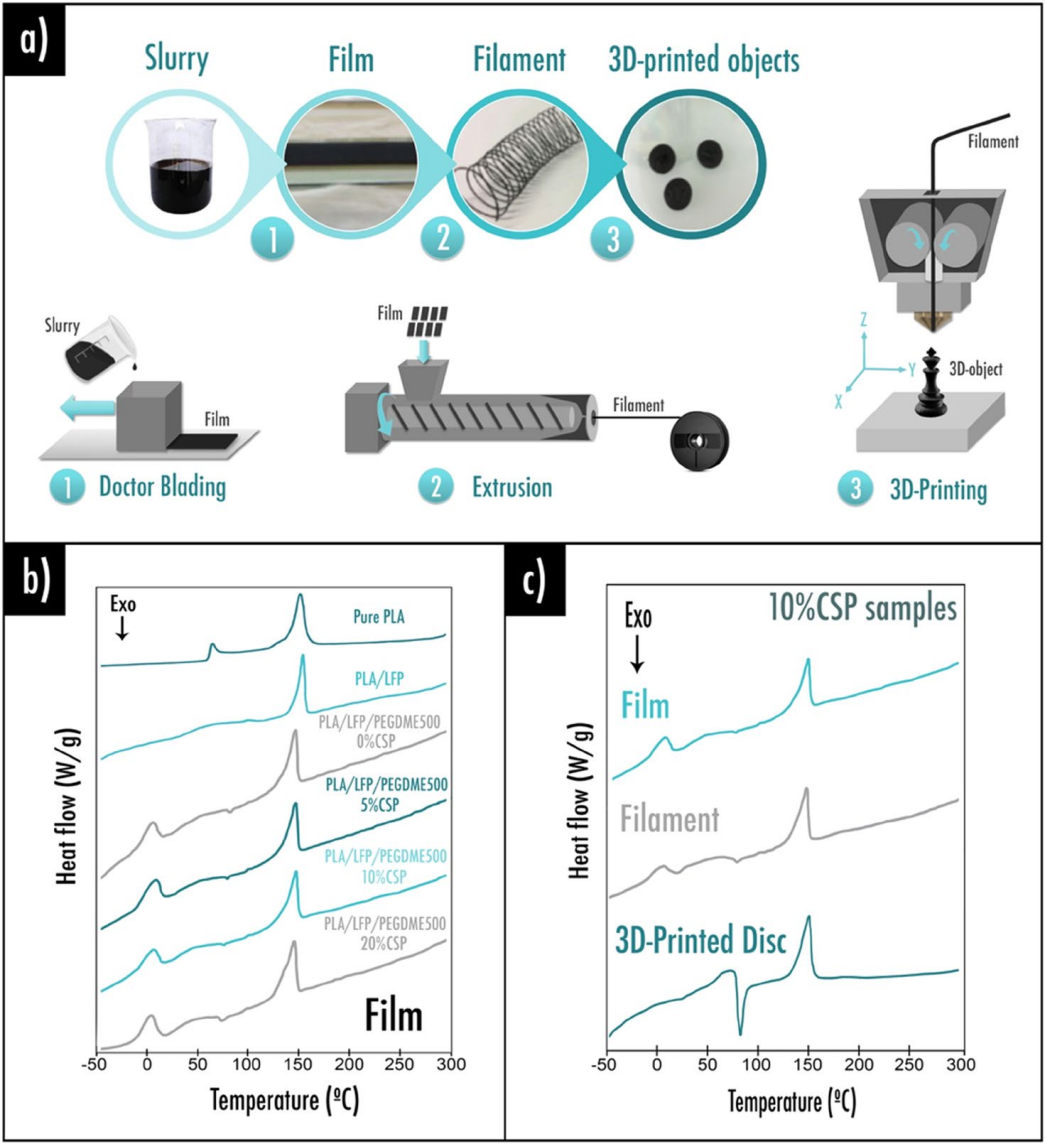
(**a**) Formulation process: (1) After mixing all of the components into a solvent, slurry is spread on a glass support by doctor blading technique and a film is finally obtained; (2) Composite film homogeneous pieces are introduced in an extruder. A typical 1.75 mm diameter 3D-printing filament is obtained and rolled; (3) Filament is introduced into a commercial FDM 3D-printer; DSC curves: (**b**) pure PLA, PLA/LFP wt% 40/60 and PLA/LFP/PEGDME500 with different amount of conductive additive (CSP); (**c**) comparison between film, filament, and 3D-printed disc for the 10%CSP sample.

The complete formulation route is exhibited in Fig. 1a. As described in our previous work^42^, the solvent method was followed to guarantee a good homogeneity at laboratory scale. Dichloromethane (DCM) was employed as it allows a quick dissolution of PLA and its relatively low boiling point leads to a rapid evaporation of the solvent after slurry doctor blading. Solvent selection is a crucial stage as it allows the active material and other additives to be evenly accommodated within the polymer matrix.

Slurry formulation for the positive electrode at increasing carbon black (CSP) concentration (Table 3) was prepared by dissolving the PLA pellets into DCM, with a weight ratio PLA/DCM 1:10, under magnetic stirring and at room temperature. Once complete dissolution of PLA was achieved, PEGDME500 acting here as plasticizer was added (40 wt.% ratio PEGDME500/PLA) and mixture was magnetically stirred for 30 minutes. In the meantime, the desired amount of active material and conductive additives were pre-mixed in a mortar before incorporation within the slurry in order to guarantee meticulous mixing (wt.% ratio PLA/AM 40:60). Again, magnetic stirring was performed during 5 h. Finally, homogeneous mixture slurries were spread onto a glass substrate following the doctor-cast technique. Self-standing composite films were achieved at room temperature after complete evaporation of the solvent overnight. Same procedure was followed for the preparation of the separator films (Table 4). After dissolution of the PLA, plasticizer was added (40 wt.% ratio PEGDME500/PLA). Then, amorphous SiO_2_ nanoparticles (average size 7 nm), display in Fig. S1, were added in the slurry and mixed until a homogeneous mixture was achieved. Subsequently, a free-standing separator film was obtained through tape-casting.

**Table 3:** Summary of the film compositions produced for the positive electrode at increasing conductive additives content (CSP). For weight-volume conversion, material densities were determined by helium pycnometer.

Sample name	Weight ratio PLA:LFP	Weight ratio PLA:PEGDME500	Weight ratio LFP:Conductive additive	Wt.% total composite PLA/LFP/Plasticizer/Conductive additive	Vol.% total composite PLA/LFP/Plasticizer/Conductive additive
**Pure PLA**	X	X	X	100/**0**/0/0	100/**0**/0/0
**PLA/LFP**	40:60	X	X	40/**60**/0/0	54/**46**/0/0
**0%CSP**	40:60	100:40	X	35/**52**/13/0	43/**37**/20/0
**5%CSP**	40:60	100:40	100:5	34/**50**/13/3	42/**36**/20/2
**10%CSP**	40:60	100:40	100:10	33/**49**/13/5	41/**35**/20/4
**20%CSP**	40:60	100:40	100:20	31/**47**/13/9	40/**34**/18/8

**Table 4:** Summary of the film compositions produced for the separator at increasing SiO_2_ nanoparticles content.

Sample name	Weight ratio PLA:SiO_2_	Weight ratio PLA:PEGDME500	Wt.% total composite PLA/SiO_2_/Plasticizer
0%SiO_2_	X	100:40	71/0/29
7%SiO_2_	100:10	100:40	66/7/27
13%SiO_2_	100:20	100:40	62/13/25
18%SiO_2_	100:30	100:40	59/18/23
22%SiO_2_	100:40	100:40	56/22/22

Figure 1b exhibits DSC plots of the composite films with various compositions of polymer, LFP, plasticizer and conductive additives. Serving here as a reference, pure PLA displays an endothermal peak related to its melting (Tm) at 146 °C and a well-defined glass transition temperature (Tg) at 63 °C. By adding LFP as active material to the PLA matrix, the endothermal peak related to the melting temperature of the film called PLA/LFP (wt% 40:60) was a little altered to lower temperature emerging at 142 °C and the Tg is not perceptible anymore. Water desorption was detected around 100 °C by the presence of a minor endothermal peak. Because of the important brittleness of the obtained composite film, a plasticizer, poly(ethylene glycol) dimethyl ether average Mn~500 (PEGDME500), was incorporated as discussed in our previous study^42^. Compared to PLA/LFP sample which only shows a clear Tm peak, a little exothermal crystallization peak (Tc) around 80 °C is induced through the incorporation of a plasticizer, in good agreement with literature^54^. This is emblematic of plasticized thermoplastics, as plasticizers may facilitate crystallinity by enhancing chain mobility. As the temperatures difference Tc-Tg is reported to be constant (~80 °C) in the whole range of plasticizer concentration^54^, the glass transition temperature (Tg) of the PLA polymer matrix should also appear in the range of the first endothermal peak at 4 °C imputed to the plasticizer PEGDME500 fusion. Plasticized PLA/LFP films display Tm inferior than without plasticizer, diminishing from 142 °C to about 132 °C.

In parallel, in order to enhance the electrical conductivity within the positive electrode, samples containing different amount of CSP (weight ratio LFP:CSP equals to 100:x with x from 5 to 20) were prepared. By increasing the CSP content, endothermal peaks at 4 °C and 132 °C, respectively corresponding to the PEGDME500 fusion and the composite film melting, remain unchanged. This behavior is however different for the exothermal crystallization peak (Tc) previously observed at 80 °C (0%CSP sample). Indeed, through the addition of CSP, crystallization peak is slightly altered to lower temperature, reaching 74 °C for the 20%CSP sample. This behavior may be explicated by the CSP presence within the PLA matrix impacting crystallinity behavior at the microscopic scale.

Extrusion and 3D-printing were concentrated on the most electrochemically-encouraging 10%CSP film sample hereafter detailed. Plots achieved from DSC for the analogous film, filament and 3D-printed disc (Fig. 1c), display similar Tm value equals to 132 °C. Nevertheless, endothermal wide peak intensity related to the PEGMDE500 fusion at 4 °C tends to drop following extrusion and 3D-printing (performed respectively at 170 and 195 °C), indicating partial plasticizer evaporation occurs during both stages. Conveniently, the lower plasticizer content still confers sufficient filament flexibility in order to feed the 3D-printer. Mechanical performances such as the Young’s modulus (223 ± 20 MPa), tensile strength (7.36 ± 0.45 MPa) and elongation at break (14.04 ± 1.35%), were determined from the stress-strain curves. As expected, those values are very low (except elongation at break) compared to pristine PLA (Young’s modulus: 3500 MPa, tensile strength: 59 MPa, elongation at break: 7%)^55^ due to the high loading of composite material here. Furthermore, after 3D-printing, the exothermal peak of crystallization area emerging at 75 °C is more intense. This behavior may be explained by the fast cooling allowed by the fans, thus leading to a less crystallized state of the polymer chains.

From our practical experiences, with a view to obtain enough mechanical performances to be printed, the total amount of charges (sum of active material and conductive additives) within the filament must not exceed a total of about 50 vol% when a plasticizer such as PEGDME500 is introduced. On the contrary, without the plasticizer introduction, the total amount of charges must not exceed about 30% otherwise the PLA-based filament will not be printable. As displayed in Table 3, in this study, the most-promising 10%CSP positive electrode filament contains 35 vol% of LFP and 4 vol% of CSP, which represents a total volume of charges equals to 39 vol% of the total composite. A compromise must be found with a view to enhance the electrochemical performances (through a high amount of charges) while still maintaining suitable mechanical properties (thanks to polymer matrix and plasticizer).

Porosities for the 10%CSP positive electrode after each step of the formulation (film, filament and 3D-printed disc) were calculated considering the complete plasticizer evaporation. While the film obtained after tape-casting originally depicts 17% porosity, it can be seen that after extrusion the filament displays only 14% of porosity. This may be easily explained by the extrusion process itself compacting the material. The 3D-printed disc displays about 29% porosity which is explicated by the successive pathways taken by the nozzle leading to a surface inhomogeneity creating high macro-porosity.

**Figure 2:**
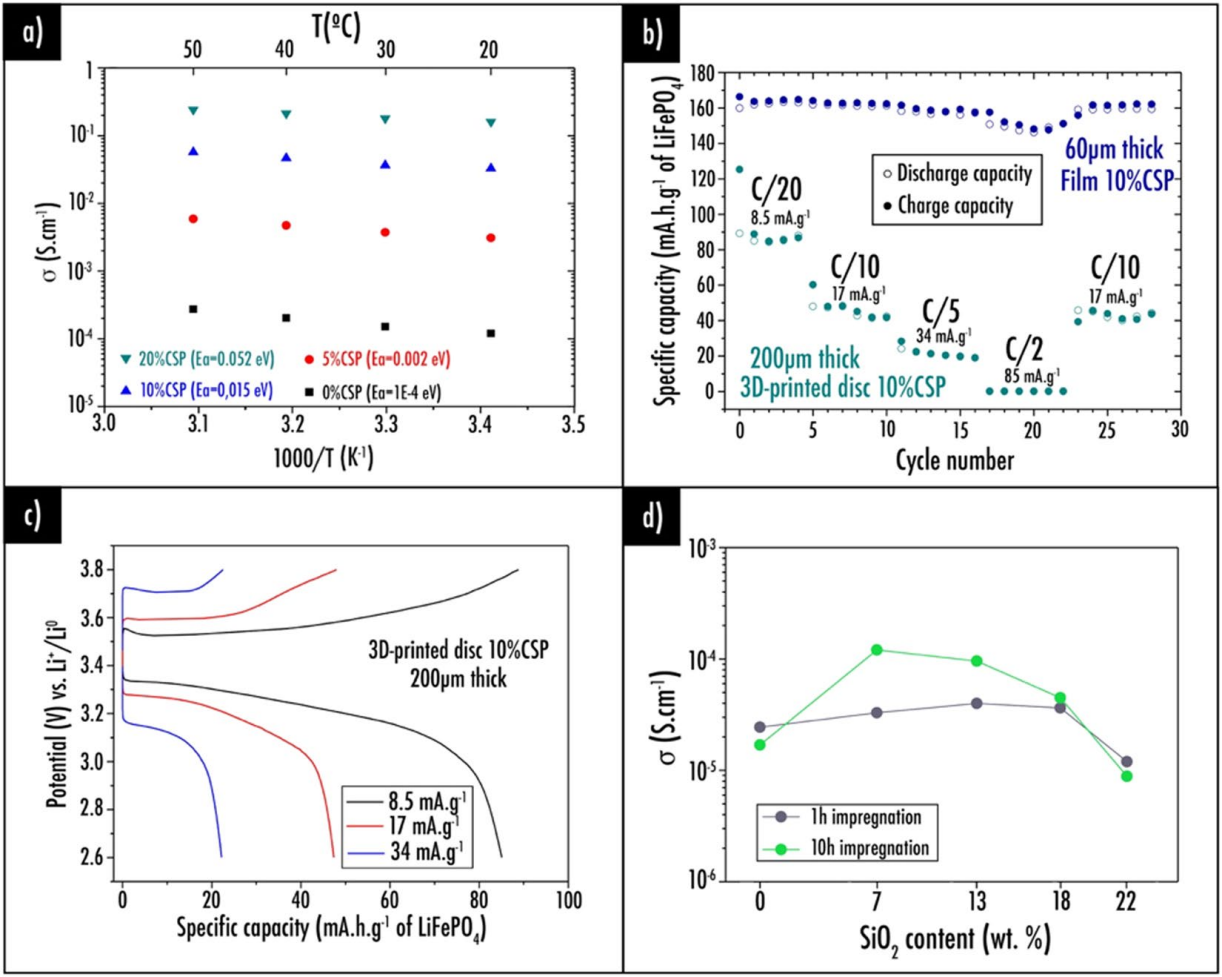
(**a**) Arrhenius plots of the electrical conductivity for samples containing CSP as conductive additives; (**b**) Capacity retention plots at different C-rate for the film and 3D-printed disc 10%CSP sample. (**c**) Charge/discharge capacity profiles for the 3D-printed disc 10%CSP sample. Note that for those experiments, a commercial fiber glass separator was used; (**d**) Conductivity after 1 h and 10 h within the electrolyte 1 M LiPF_6_ in EC:DEC 1:1 vol% for samples containing different SiO_2_ content.

The effect on electronic conductivity of carbon black, CSP, was studied. For samples obtained after slurry casting and labeled in Table 3 (weight ratio LFP:CSP equals to 100:x with x from 5 to 20), specific impedance spectra were investigated at temperatures ranging between 20 °C to 50 °C. The achieved Nyquist and Bode curves are in accordance with an electronic conductor classical behavior as Nyquist plot portrays only Z’ real part while |Z| magnitude is remained steady for all frequencies. The electrical conductivity, for all samples of equal thickness and area, is rising with temperature as exhibited in Fig. 2a and activation energy values varying between 0.0001 eV and 0.052 eV were estimated. Panabière *et al*.^56^ showed that electrons transport mechanism in carbon/polymer mixtures used in battery field was tunneling with no temperature dependence (metal-like behavior). Nonetheless, such temperature dependence would be perceived when strong dipoles are adsorbed at the carbon particles surface. In this respect, it is not surprising to observe activation energy values increasing with higher CSP content. Conductive additive contributes to greater conductivity values compared to the 0%CSP sample without additives, which is in good agreement with what was reported by Guy *et al*.^57^ However, here, a higher loading of CSP is needed to reach similar values^57^, which is undoubtedly due to the high quantity (32.8 wt%) of the polymer matrix (PLA) having a detrimental effect on the homogenization and CSP distribution which may be isolated. Amongst the various film samples tested, 20%CSP exhibits the greatest values of electrical conductivities. Nevertheless, it is worth noticing that conductivity values are still relatively low due to the LFP insulator behavior (enhanced up to 0.16 S.cm^−1^ for the 20%CSP sample at 20 °C) as compared to values obtained in our previous study^42^ for the graphite-based negative electrode (0.40 S.cm^−1^ at 20 °C). Carbon coating is therefore important when LFP is considered as active material^58^. Here, the incorporation of carbon additives such as CSP is essential when considering exotic composite compositions with such an important amount of PLA polymer matrix.

On the other hand, a thorough characterization study was performed to establish the electrochemical behavior of the two most electrically conductive film samples 10%CSP and 20%CSP (Figs. 2b and S2). Note that commercial glass fiber was used as separator for the following experiments. For each sample of the same size (60 μm-thick and 11 mm diameter, surface equal to 0.950 cm^2^) acquired after doctor blading (described in Table 3), the potential profiles versus specific capacity based on the active material and specific capacity versus cycle number were examined at diverse current densities (8.5, 17, 34, and 85 mA g^−1^ of active material equivalent to C/20, C/10, C/5, and C/2). Among those samples, it was shown that 10%CSP film displayed the best electrochemical performances reaching specific capacity value up to 165 and 162 mAh.g^−1^ of active material at C/20 and C/10 respectively. It appears that 20%CSP film displays slightly inferior capacity values. Besides, this latter composition was discarded as CSP tends to agglomerate thus affecting the sample homogeneity. As theoretical capacity was almost reached with the 10%CSP sample and as no homogeneity issues were observed, it was consequently decided to focus our studies on this optimized sample for the positive electrode. Consequently, the corresponding 10%CSP filament (756 mg of active material per cm^3^ of composite) was produced and 200µm-thick 3D-printed discs were characterized electrochemically (Fig. 2b,c). Compared to specific capacity values obtained for the latter 10%CSP 60µm-thick film, values displayed for the corresponding 200µm-thick 3D-printed disc were observed to be much lower: 87 mAh g^−1^ of active material at C/20 (equals to 43 mAh g^−1^ of the total composite or also 66 mAh cm^−3^ considering the total volume of the electrode) and 45 mAh g^−1^ of active material at C/10 (representing 22 mAh g^−1^ of the total composite or also 34 mAh cm^−3^). This important gap is due to the difference of thickness between the film (60 µm) and the 3D-printed disc (200 µm) induced by the 3D-printer low thickness resolution on the first layer. 3D-printed disc shows an irreversible capacity during the first cycle of 38 mAh g^−1^ of active material, equivalent to a percentage loss of 42% at a current density of 8.5 mA g^−1^ (C/20).

Thereafter, it was decided to check the electrochemical performances of the assembled complete system by using both 200µm-thick optimized 3D-printed electrodes. Hence, sample 10%CSP aforementioned, containing LFP as active material, was used as positive electrode while the 3D-printed graphite electrode optimized in our previous study^42^ was used as negative electrode. In this latter, as it can be seen in Fig. S3 depicting the microstructure of both 3D-printed electrodes observed by SEM, the CSP particles are evenly dispersed within the polymer matrix. Here, the homemade PLA/SiO_2_ separator was used instead of the commercial fiber glass mentioned earlier. It was subjected to ionic conductivity and swelling measurements after electrolyte soaking with various amount of silica.

Indeed, introduction of ceramic fillers within the polymer matrix has attracted substantial attention in literature^59^, due to their ability in improving thermal stability, mechanical strength as well as ionic conductivity of the separators. In order to enhance those last parameters, the nanosilica content was optimized from 0 to 22 wt.%. For each separator sample described in Table 4, specific impedance spectra were investigated at 25 °C under argon after immersion within the electrolyte during 1 h and 10 h (Fig. 2d). First of all, by introducing SiO_2_ nanoparticles within the PLA polymer matrix, it was shown that swelling percentage tends to decrease. Indeed, after being immersed 10 h within the electrolyte, 22%SiO_2_, 18%SiO_2_, 13%SiO_2_, 7%SiO_2_ and 0%SiO_2_ samples depicted a swelling percentage equals to +2%, +4%, +5%, +8% and +14% respectively. Moreover, after 1 h immersed within the electrolyte, conductivity was improved up to 3.96 × 10^−5^ S.cm^−1^ for the 13%SiO_2_ sample compared to value reaching only 2.41 × 10^−5^ S.cm^−1^ for sample 0%SiO_2_. After 10 h immersion, the most conductive sample was 7%SiO_2_ displaying 1.20 × 10^−4^ S.cm^−1^, thus corresponding to seven times the conductivity value obtained for the reference 0%SiO_2_ (only 1.67 × 10^−5^ S.cm^−1^). An explanation of this behavior is that composite separators have higher electrolyte uptake due to the SiO_2_ particles introduction improving wettability compared to the pristine separator^60^. Considering the complete plasticizer evaporation, the optimized sample “7%SiO_2_” presents 11.3 mol% of SiO_2_ (9.6 wt%), close to the optimized silica amount of 15.7 mol% (4 wt%) reported by Caillon-Caravanier *et al*.^61^ Through the SiO_2_ addition, a more porous structure is formed, thus contributing to faster electrolyte uptake. As also described by Caillon-Caravanier *et al*.^61^, it is worth mentioning that a too high content of SiO_2_ can have a detrimental effect on ionic conductivity as it is clearly visible for the 22%SiO_2_ sample (8.71 × 10^−6^ S.cm^−1^ after 10 h). When maximum porosity is reached, further addition reduces absorption ability and conductivity by limiting the porous volume and by interaction of some fraction of the lithium cation with silica^62^. Thus, it was decided to use the optimized 7%SiO_2_ sample as separator for the following experiments.

**Figure 3:**
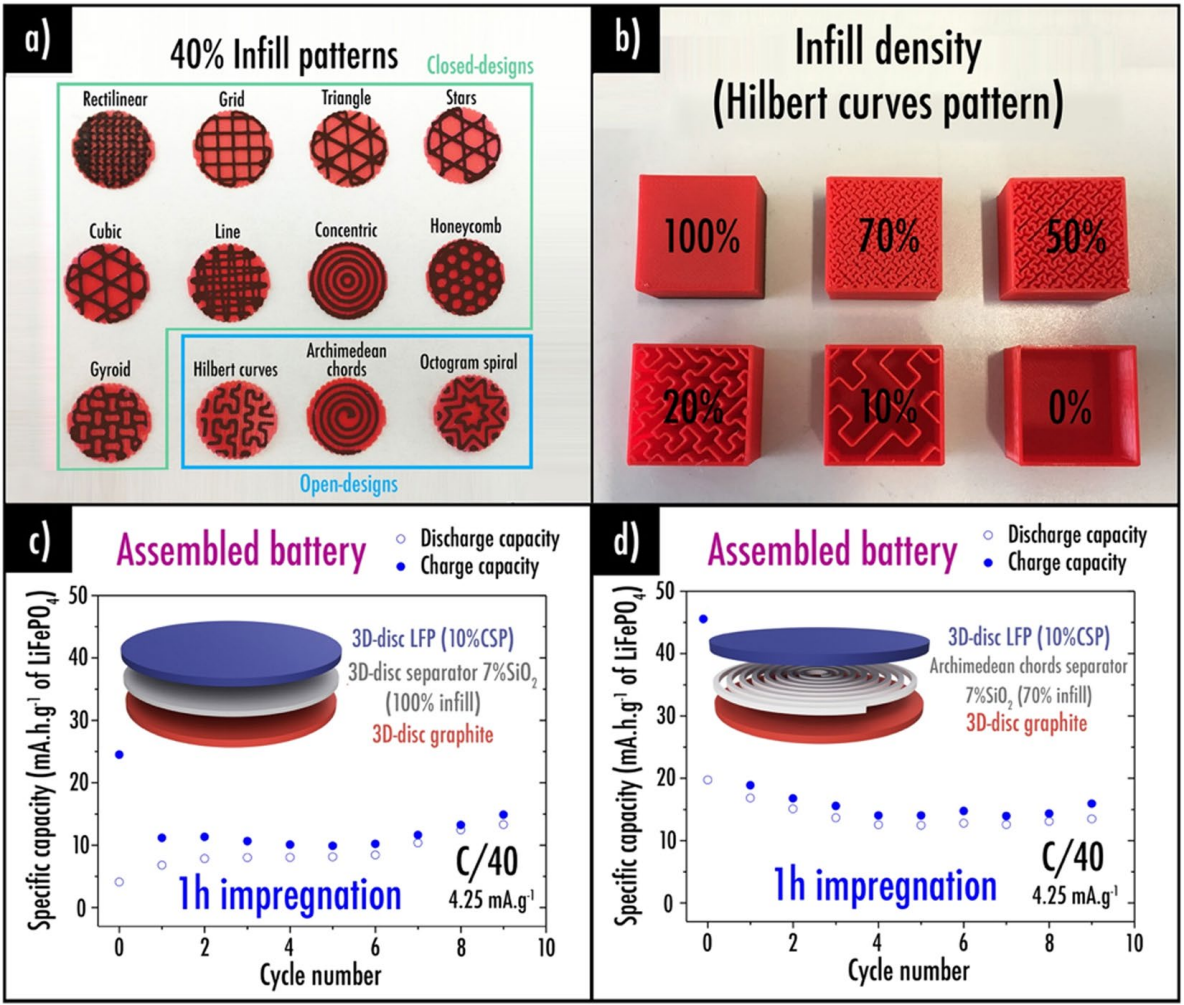
(**a**) Different separator infill patterns that can be obtained by using classic 3D-printing slicer software (40% infill density); (**b**) Various infill densities of the same infill pattern (Hilbert curves); Capacity retention plots at 4.25 mA.g^−1^ (C/40) for the complete assembled battery after 1 h impregnation: (**c**) using 100% infill density separator and (**d**) using a 70%infill density Archimedean chords pattern. Here, note that each layer is 200 µm thick.

With a view to favor the liquid electrolyte uptake within the system, we also decided to take advantage of the new design capabilities conferred by 3D-printing. Indeed, various separator infill patterns (Fig. 3a) can be easily obtained (automatically generated) by using classic 3D-printing slicer software. One can easily understand that open-designs such as the Hilbert curves, Archimedean chords or Octogram spiral will favor the spread of liquid electrolyte compared to closed-designs (rectilinear, grid, triangle, stars, cubic, line, concentric, honeycomb and gyroid). Moreover, in order to avoid short circuit, next step is to set the infill density properly (measured in percent). A print with low infill density (Fig. 3b) for the separator will favor the electrolyte uptake while it will also favor short-circuits. Thus, a compromise must be reached.

Regarding the assembled complete system, Archimedean chords was chosen among the open-designs as it is the only pattern that can be printed in one fragment and thus be manipulated. Furthermore, infill density was set to 70% as it seems enough to avoid short-circuits. The capacity retentions at 4.25 mA g^−1^ (C/40) after 1 h impregnation were plotted. As reference, capacity retention for the system using 100% infill density separator disc is displayed in Fig. 3c. Good capacity retention is observed after 1 h impregnation with the 70% Archimedean chords pattern for the separator as it enables faster liquid electrolyte uptake (Fig. 3d). On the contrary, sample with 100% infill density separator disc, displays increasing capacity after 1 h during the first cycles. It is worth mentioning that a very low reversible capacity value of about 15 mAh g^−1^ of active material was obtained during 10 cycles at 4.25 mA g^−1^ (C/40). Those poor performances are induced by the 3D-printer first layer thickness limitation as it is not possible to obtain <200 µm electrodes and separator. Thus, balancing of the cell was not possible.

**Figure 4:**
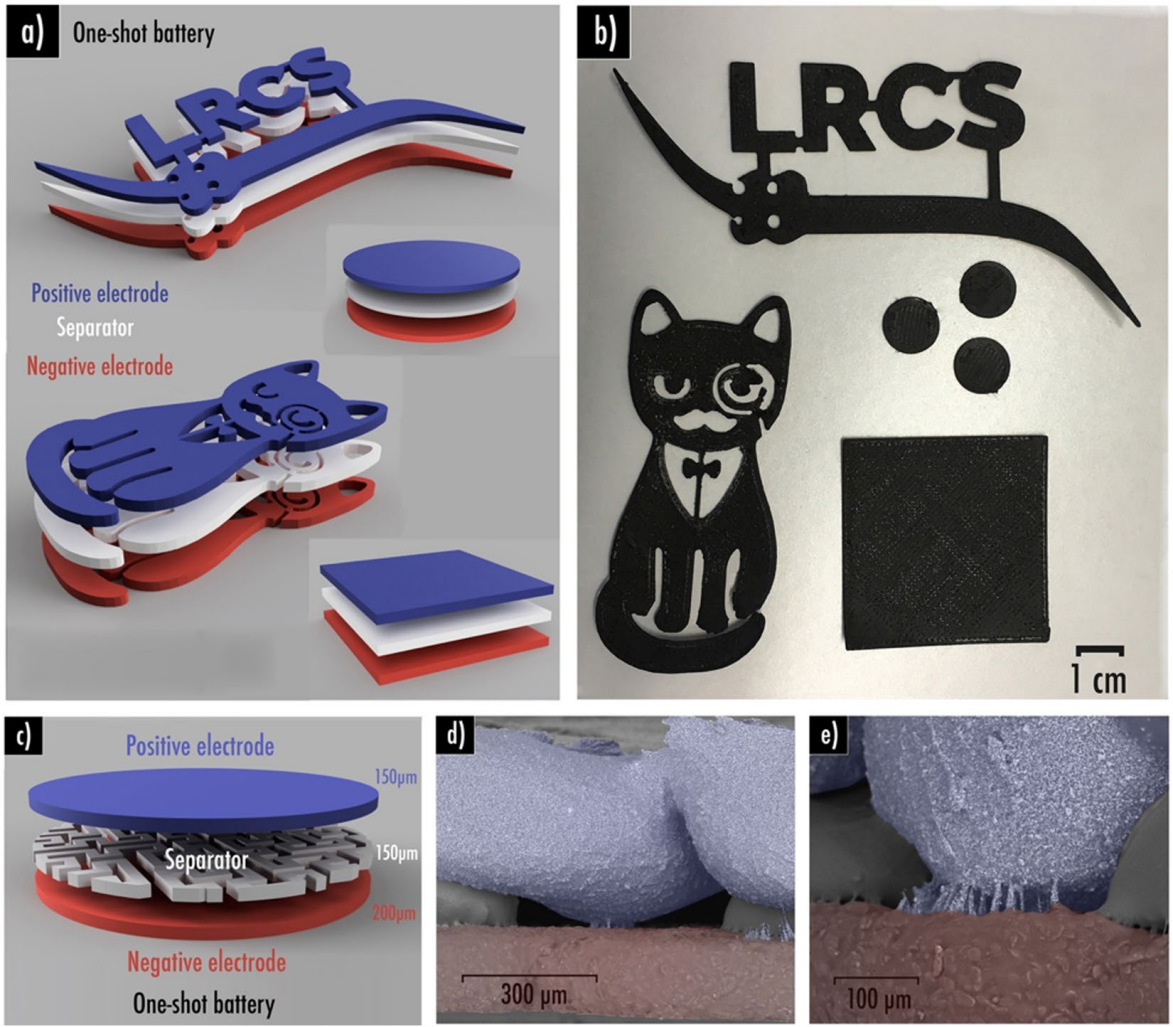
Complete “one-shot” lithium-ion battery of any shape can be easily (**a**) designed and (**b**) 3D-printed; (**c**) Scheme of the 3D-printed in “one-shot” lithium-ion battery using Hilbert curves pattern 70% infill density as separator layer (150 µm thick). (**d,e**) Backscattered electron SEM images of the short-circuits observed within this system. It is important to note that 7%SiO_2_ and 10%CSP sample were respectively used here as separator and positive electrode while our previously reported (cf. ref. ^42^) Graphite/PLA/CSP/PEGDME500 filament was used to print the negative electrode.

Finally, as reported for the first time by Reyes *et al*.^19^, we confirmed the printability of the complete battery in a single print that we shall refer to as “one-shot”. Complete lithium-ion battery of any shape can be easily printed such as disc, square, our laboratory logo or even a fancy cat (Fig. 4a,b). This was technically possible by a succession of numerous important steps which are described in more details in the experimental part. Filaments were loaded one after another by pausing the printer. Cleaning steps of the nozzle were unavoidable to ensure good homogeneity. Note that each layer was perpendicularly printed to the precedent to favor adhesion. These tedious steps might be simplified in the future by using a multi-nozzle 3D-printer. However, this solution also comes with drawbacks as nowadays the x-y and z calibrations are still difficult and retraction does not always work properly. Filament leftovers can get stuck on the print when the extruder moves and thus leading to eventual short-circuits.

Here again, in order to favor the liquid electrolyte uptake within the “printed in one-shot” system, we also decided to set an infill pattern for the separator (Fig. 4c). Among the open-designs, Hilbert curves seems to be the most promising as this is the only pattern enabling liquid electrolyte to enter using different paths. Indeed, Archimedean chords and Octogram spiral display only one entry for the electrolyte. Short-circuits are more complicated to avoid in the “one-shot” configuration as printing is performed at 195 °C with this specific 3D-printer to prevent under-extrusion (when not enough material from the filament is extruded during a print, resulting in gaps or missing layers that strongly prejudice the print quality). Indeed, even with a 70% infill density separator (150 µm thick), the above layer corresponding to the positive electrode have tendency to collapse naturally by gravity to fill the gaps created by the separator pattern, thus leading to short-circuits. This phenomenon is easily observable by scanning electron microscopy (Fig. 4d,e).

**Figure 5:**
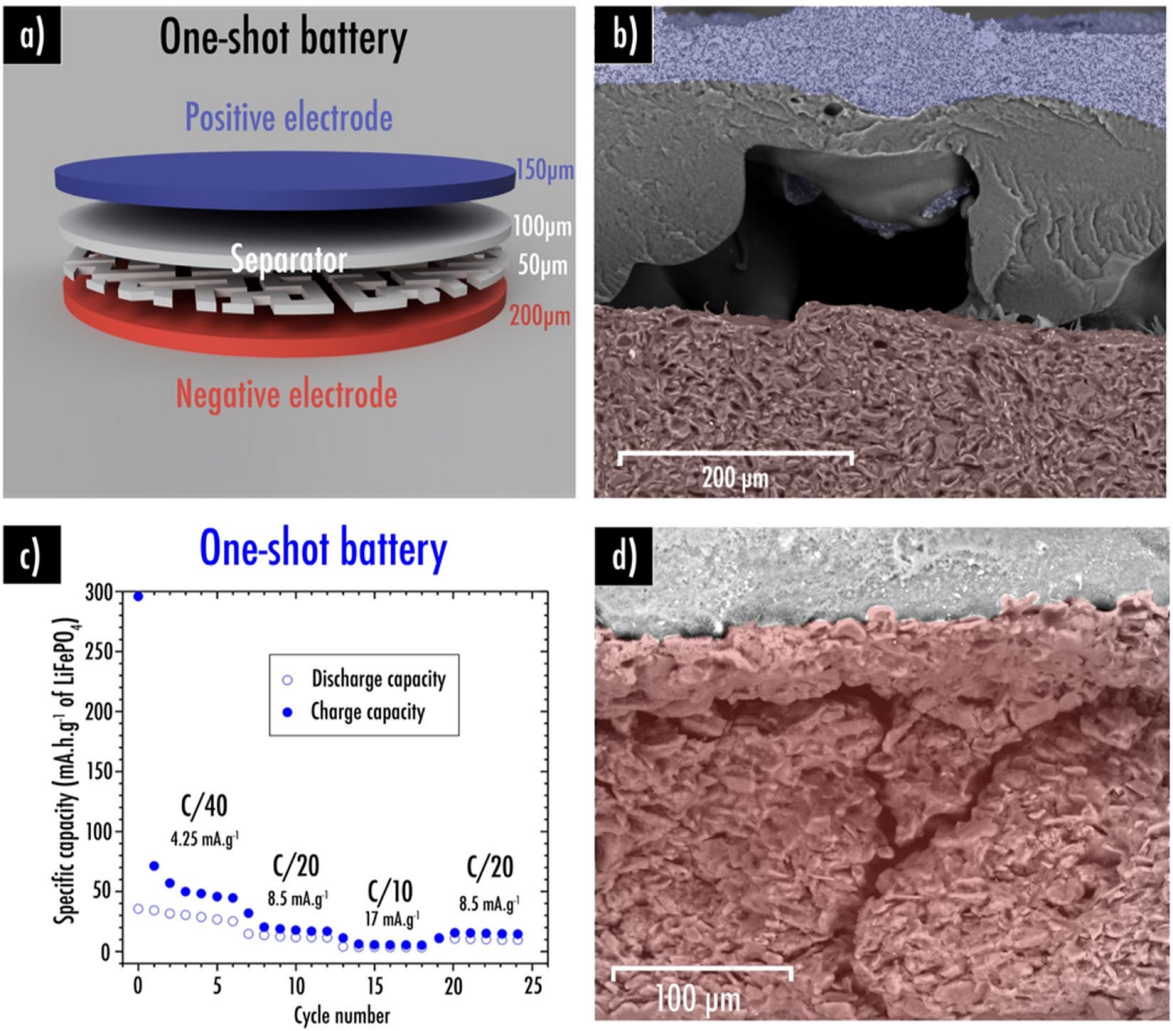
(**a**) Scheme of the 3D-printed in “one-shot” lithium-ion battery using Hilbert curves pattern 70% infill density as first separator layer (50 µm thick) and 100% infill density (100 µm thick) as second separator layer; (**b**) Backscattered electron SEM image of the system; (**c**) Capacity retention plots at different C-rate for the 3D-printed in “one shot battery”; (**d**) Backscattered electron SEM image of the cracks observed within the negative electrode after cycling. It is important to note that 7%SiO_2_ and 10%CSP sample were respectively used here as separator and positive electrode while our previously reported (cf. ref. ^42^) Graphite/PLA/CSP/PEGDME500 filament was used to print the negative electrode.

To prevent this behavior, it was decided to add a 100% infill density separator layer (100 µm) above the 70% infill density Hilbert curves pattern (50 µm) as displayed in Fig. 5a. Narrow caves created by the separator pattern can be observed by SEM/EDS (Fig. 5b). Ceiling of those cavities is homogeneously covered by a separator PLA-SiO_2_ thin layer thus preventing any contact between both electrodes (Fig. S4). Noteworthy that this uppermost separator layer might also prevent lithium dendrite propagation while subjected to improper cycling conditions as low temperature and high rate.

Figure 5c depicts the capacity retention plots at different C-rates for the one-shot fully 3D-printed disc system. In this configuration, as separator thickness diminution as well as balancing of the cell are now possible, slightly higher reversible capacity values were reached (30 mAh g^−1^ of active material at C/40, corresponding to 15 mAh g^−1^ of the total composite or also 6.5 mAh cm^−3^ considering both electrodes and separator total volume) compared to the assembled cell (15 mAh g^−1^ of active material at C/40). After cycling, it is worth mentioning that cracks were observed within the negative electrode layer (Fig. 5d). It is well known that the volume expansion ΔV/V is equal to the trace of the strain tensor $\varDelta {\rm{V}}/{\rm{V}}=\mathrm{Trace}\,(\epsilon )=3.{{\rm{\varepsilon }}}_{{\rm{xx}}}$ with ε_xx_ the extensional strain along the coordinate axe x equal the two others under the assumption of isotropic swelling^63^. Knowing that graphite exhibits a volume expansion up to 10% when lithiated leading to a strain ${{\rm{\varepsilon }}}_{{\rm{xx}}}\cong 3.3 \%$, higher than the elongation at break closed to 3% obtained from the stress-strain curve of the negative electrode. On the contrary, as expected, this phenomenon is not observed for the positive electrode as it starts to shrink because of the lithium departure upon first charge.

With a view to improve the overall system, next step would be to develop a current collector filament in order to print the first layer subjected to the 200 µm thickness limitation. Hence, it would be possible to print thinner (down to 50 µm) and balanced electrodes and thus further improve the electrochemical performances. On the other hand, graphite granulometry could be decreased to favor the particles dispersion within the polymer matrix, better accommodate the volume constraints and thus prevent cracks upon cycling.

## Conclusion

3D-printable PLA/LFP and PLA/SiO_2_ filaments, specifically conceived to be used respectively as positive electrode and separator in a Li-ion battery were produced to feed an FDM 3D printer. As reported in our previous study^42^, thanks to plasticizer PEGDME500 addition, the active material content within the positive electrode filament was increased as high as possible to improve the electrochemical performances while still providing enough mechanical properties to be printed. Films containing CSP showed higher electrical conductivity and specific capacity, indicating their addition enables several isolated active material particles to be electronically connected to the percolating network. Because theoretical capacity was almost reached with the optimized 10%CSP film sample, its corresponding filament was employed as material source for the 3D-printer. Furthermore, the impact of ceramic additives (SiO_2_) on ionic conductivity in the separator was studied. Samples with various amount of silica were subjected to ionic conductivity and swelling measurements after electrolyte soaking. After 10 h impregnation, the most conductive sample was 7%SiO_2_.

For the first time, considering both optimized filaments composition and using our previously reported PLA/graphite filament^42^ for the negative electrode, assembled and printed in “one-shot” complete LFP/graphite battery cells were 3D-printed by means of FDM.

Taking advantage of the new design capabilities conferred by 3D-printing, separator patterns and infill density were considered with a view to enhance the liquid electrolyte impregnation and avoid short-circuits. Archimedean chords and Hilbert curves appear to be the most promising patterns to be used respectively within the assembled and the “one-shot” configurations. Infill density values >70% would be studied in more details in future with a view to prevent short-circuits. While low reversible capacity values were reached for the “one shot” system (30 mAh g^−1^ of active material at C/40, corresponding to 15 mAh g^−1^ of the total composite or also 6.5 mAh cm^−3^ considering both electrodes and separator total volume), it is important to understand that separator thickness diminution as well as balancing of the cell were now possible in this configuration, thus improving the performances compared to the assembled cell (15 mAh g^−1^ of active material at C/40).

This study, by merging both battery and 3D-printing technologies, addressed numerous electrochemical (thickness, electronic and ionic conductivity, electrolyte uptake) and 3D-printing parameters (infill density, infill pattern, perimeters, over and under-extrusion, retraction), and opens the way for a better performing 3D-printed lithium-ion battery.

Finally, as this work acts here as proof of concept, authors are well-aware that for now electrodes and separator patterns are 2D and thus achievable using non-3D printing techniques. Future work, however, would be concentrated on complex 3D-battery architectures that necessitates significant system adjustments and a thorough design optimization. Upcoming research may also be dedicated to mechanically ameliorate the FDM 3D-printer resolution and simplify the tedious steps to print the full-battery in one-shot by using a multi-nozzle configuration.

## Methods

### Materials

Polylactic-acid (PLA 4032D) pellets were provided by NatureWorks, USA. Dichloromethane (DCM) was supplied by VWR Chemicals, USA. Timcal TIMREX® SLS graphite (SSA: ~1.5 m^2^ g^−1^, particle size: 15 µm) was used as active material for the negative electrode of the lithium-ion battery while Ales LFP (particle size: 2 µm) was used as active material for the positive electrode. Poly(ethylene glycol) dimethyl ether average Mn~500 (PEGDME500) was supplied by Sigma-Aldrich, USA. Carbon black Timcal Super-P (CSP), (SSA: 62 m^2^ g^−1^) and SiO_2_ nanopowder (diameter: 7 nm) were supplied by Sigma-Aldrich, USA.

### Filaments formulation

An extruder Filabot Original provided by Filabot Triex LLC, USA was fed consistently with 3 mm × 3 mm composite film fragments in order to obtain classical 1.75 mm diameter 3D-printing filaments. Extruder temperature was set about 35 °C higher than the melting temperature of the composite film determined from DSC. The filament released from the extruder nozzle was subsequently rolled around a spool by using a Filabot spooler (Filabot Triex LLC, USA). The extruder was purged thoroughly with pure PLA before extrusion of each sample. Electrode filaments were subsequently kept in appropriate storage conditions under low temperature to prevent plasticizer evaporation and in confined environment with low humidity. Well aware of the plasticizer evaporation upon time, they were almost immediately (one week of storage maximum) used as material source for the 3D-printer.

### Printing

11 mm diameter separator, positive and negative electrode discs (200 µm thick) and elaborated 3D structures were printed by using a Prusa MK3 3D-printer (Prusa Research, Czech Republic). Nozzle standard input and output diameters of respectively 1.75 mm and 0.4 mm were used. Best resolution in the Z direction is 0.20 mm for the first layer and 0.05 mm for the following. Nozzle temperature was set with this specific printer to 60 °C above the Tm of the composite filament deduced from DSC as fan settings was set to 100%. Bed temperature was set to 60 °C in order to enhance the adherence of the first printed layer. The external vertical shells perimeter setting was set to 0 to favor the electrolyte spread. Prior printing of each sample, nozzle was cleaned thoroughly by printing a 2 cm^3^ purge cube with the corresponding filament. The printability of the complete battery in a single print (“one-shot”) was technically possible by a succession of numerous important steps: print the first 200 µm layer with the optimized negative electrode filament, put the printer in pause, remove the negative electrode filament, load the separator filament, ensure it is well loaded, make sure nozzle is cleaned with the separator filament, print layers of separator above the precedent negative electrode, put the printer in pause, remove the separator filament, load the optimized positive electrode filament (10%CSP), ensure it is well loaded, make sure nozzle is cleaned with the corresponding filament, print layers of positive electrode above the precedent separator layer.

### Differential scanning calorimetry (DSC)

Thermal studies of films, filaments and 3D-printed discs were carried out by differential scanning calorimetry on a DSC204F1 supplied by NETZSCH-Gerätebau GmbH, Germany. All tests were performed at a heating rate of 10 °C min^−1^, between −60 to 300 °C in argon atmosphere (50 mL min^−1^), using about 10 mg of sample of each composition. Heat flow data from the first heating operation was recorded.

### Electron microscopy

The general homogeneity and the inner material dispersion within the PLA matrix of the produced composite filaments was examined by making use of a FEI Quanta 200F (Thermo Fisher Scientific, USA) scanning electron microscope (SEM) in high vacuum mode. The secondary and backscattered images were recorded with a 5 kV acceleration voltage. Energy Dispersive X-ray Spectroscopy (EDS) elementary maps (Fe, P, Si, C) for the “one-shot” sample were recorded at 20 kV. Selected Area Electron diffraction (SAED) patterns and transmission electron microscopy (TEM) images were obtained with a FEI Tecnai G2 electron microscope operated at 200 kV. Samples were prepared by dispersing the powder in ethanol and depositing a drop of the suspension onto a holey carbon copper grid.

### Mechanical characterization

As described in our previous work^42^, following solvent casting, the composite films were compression molded into 2 mm thick plates using a Doloutes hydraulic press at 160 °C during 2 minutes prior cooling down to room temperature. Afterwards, samples were cut into specimens with a punch as per ISO 527 standard for additional mechanical characterization (tensile testing). The tensile mechanical experiments were accomplished on a Llyod LR 50 K tensile machine set with a 1 KN load cell. Tests were controlled by displacement with a speed of 10 mm min^−1^. Five samples of each composition (dumb bell shaped) were tested and the average values are reported.

### Electrical conductivity characterization

Electrochemical impedance spectroscopy tests were done by using a MTZ-35 frequency response analyzer and an intermediate temperature system (ITS) supplied by BioLogic, France. It is worth noting that the exact same procedure than the one reported in our anterior work was respected^42^. Indeed, inductive phenomenon from the cables were avoided by performing system calibration of the empty ITS with cables. Therefore, only sample signal was measured subsequently. 200 µm thick films were punched into 5.50 mm diameter discs. The latter were introduced into a controlled environment sample holder (CESH) to perform AC impedance under air at temperatures varying from 20 °C to 50 °C (upon heating in steps of 10 °C). An excitation voltage of 0.01 V and a frequency range of 0.2 MHz to 1 Hz (20 points per decade and 10 measures per points) were applied here. Film electronic conductivities and activation energy were deduced from the Nyquist and Phase-Bode plots of the complex impedance. Conductivities were calculated from the Eq. 1:

1 $$\sigma =\frac{1}{R}\times \frac{d}{A}$$

where d is the pellet thickness, A is the pellet surface area, and R is the respective resistances determined from the Nyquist and Bode plots.

### Electrochemical characterization

Inside an argon filled glovebox (H_2_O < 0.1 ppm, O_2_ < 0.1 ppm), coin cells were assembled. Metallic lithium was used as counter/reference electrode for half cells while samples as working electrode. Fiber glass separator was provided by Whatman, GE Healthcare, USA. 150 µL of 1 M LiPF_6_ in ethylene carbonate and diethyl carbonate (EC-DEC 1:1 weight ratio) was used as electrolyte and supplied by Merck KGaA, Germany. Cells were galvanostatically discharged (lithiation) and charged (delithiation) at different current densities calculated per gram of active material, between 3.8 and 2.6 V (vs Li/Li^+^) by means of a BCS-805 (BioLogic, France). Electrochemical tests were performed at 20 °C.

## Supplementary information

Figure S1, Figure S2, Figure S3, Figure S4
