# Supplementary material for: Three-Dimensional Printing of a LiFePO4/Graphite Battery Cell via Fused Deposition Modeling
Source: Sci Rep. 2019 Dec 2;9:18031. doi: 10.1038/s41598-019-54518-y (PMC6888866; doi:10.1038/s41598-019-54518-y)
Supplement: Supplementary file 1 — Figure S1, Figure S2, Figure S3, Figure S4 [file 41598_2019_54518_MOESM1_ESM.pdf]

Supporting Information for

Three-Dimensional Printing of a $LiFePO_4$/Graphite Battery Cell via Fused Deposition Modeling

Alexis Maurel[*,1,2,3], Sylvie Grugeon[1,3], Benoit Fleutot[1,3], Matthieu Courty[1,3], Kalappa Prashantha[4,5], Hugues Tortajada[2], Michel Armand[1], Stéphane Panier[2] & Loic Dupont[*,1,3,6]

[1] Laboratoire de Réactivité et de Chimie des Solides, UMR CNRS 7314, Hub de l'Énergie, Université de Picardie Jules Verne, 33 rue Saint Leu, 80039, Amiens Cedex, France

[2] Laboratoire des Technologies Innovantes, LTI-EA 3899, Université de Picardie Jules Verne, 80025 Amiens, France

[3] RS2E, Réseau français sur le stockage électrochimique de l'énergie, FR CNRS 3459, 80039 Amiens Cedex, France

[4] IMT Lille Douai, Institut Mines-Télécom, Centre d'Enseignement, de Recherche et d'Innovation (CERI): Matériaux et Procédés Innovants, 941 rue Charles Bourseul C.S.10838, 59508 Douai Cedex, France

[5] Université de Lille, 59000 Lille, France

[6] Plateforme de Microscopie Électronique (PME) de l'Université de Picardie Jules Verne, Hub de l'Énergie, 15 rue Baudelocque, 80000 Amiens, France

* alexis.maurel@u-picardie.fr
* loic.dupont@u-picardie.fr

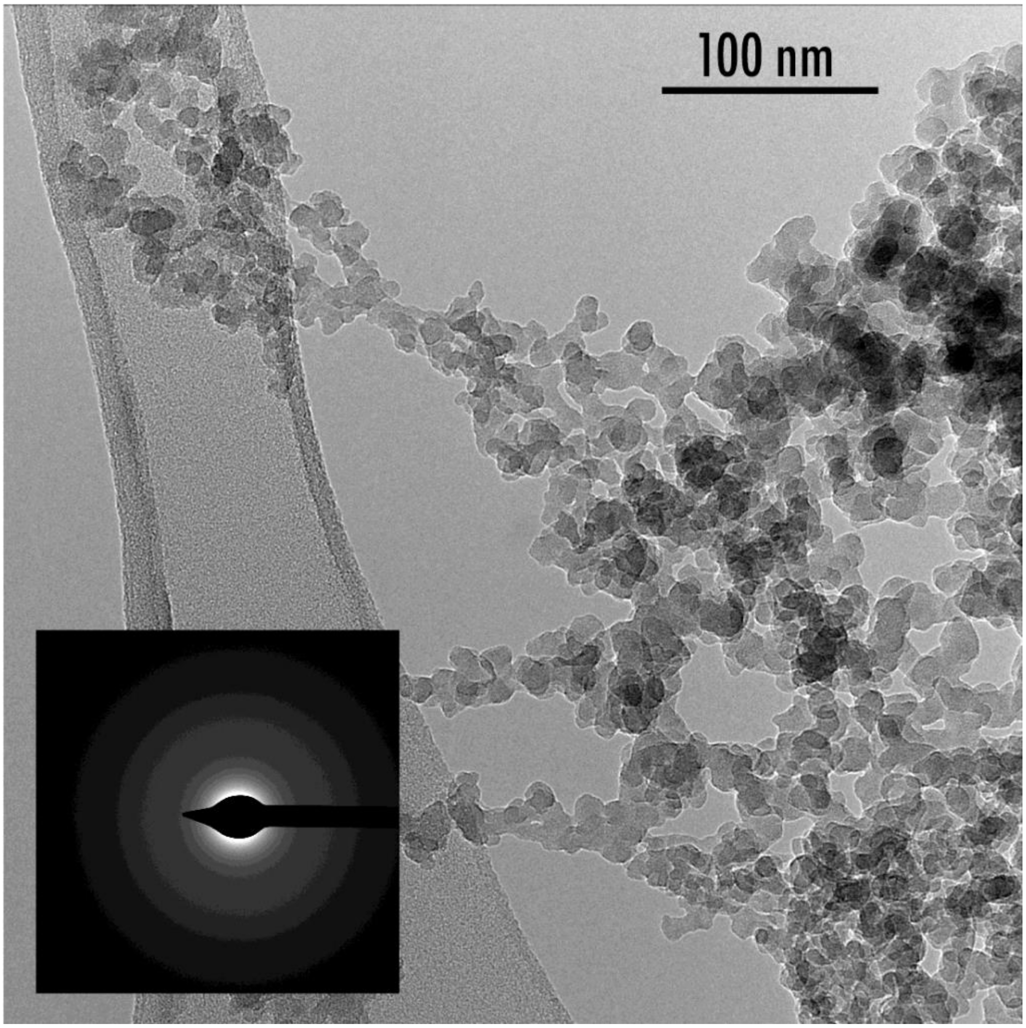

Figure S1. TEM image of the amorphous SiO_2 particles. Selected area electron diffraction (SAED) pattern is displayed in inset.

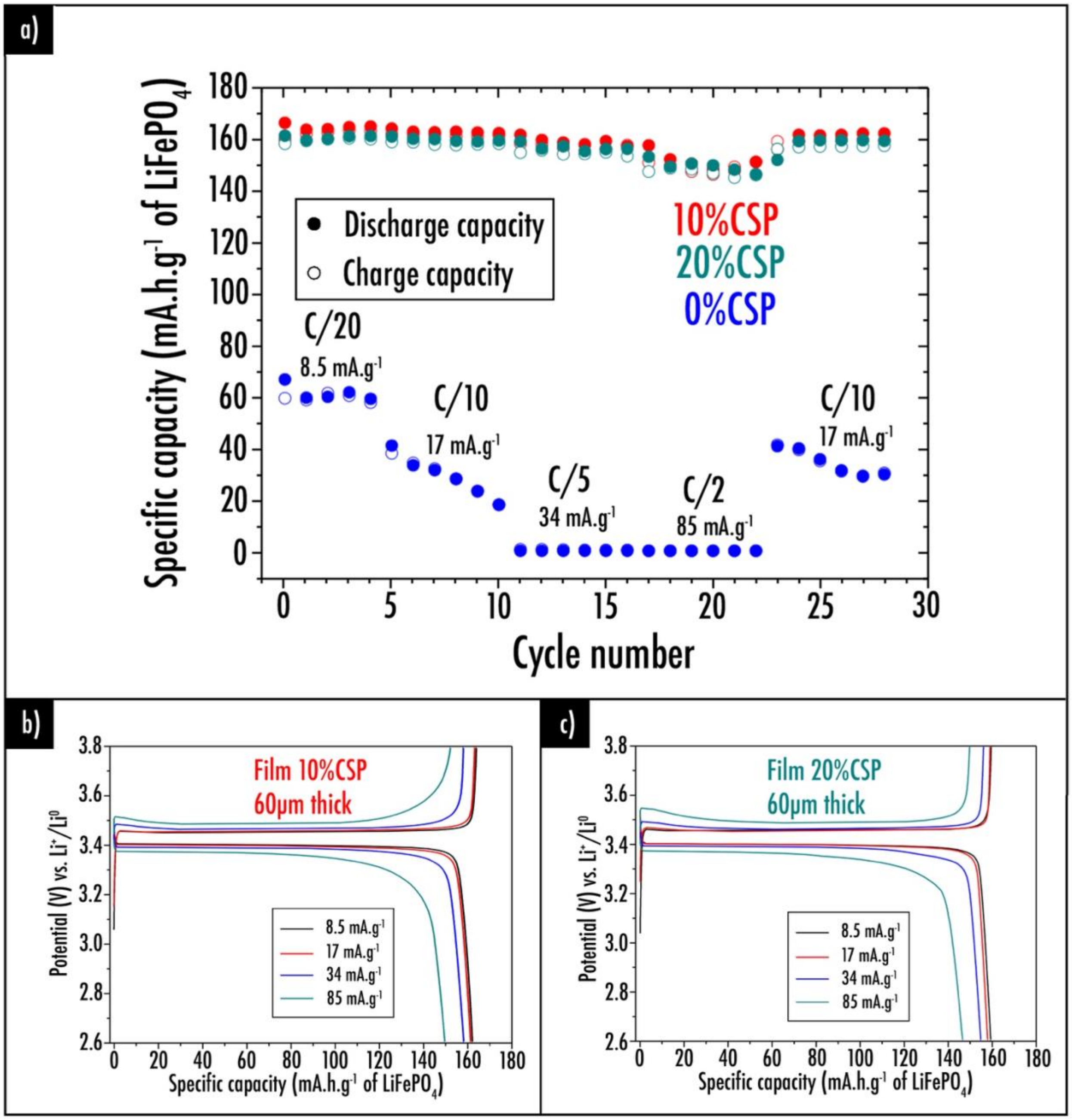

Figure S2. (a) Capacity retention plots at different C-rate for the 0%CSP, 10%CSP and 20%CSP positive electrode films; Potential profiles versus specific capacity based on the active material for (b) the 10%CSP and (c) the 20%CSP film samples.

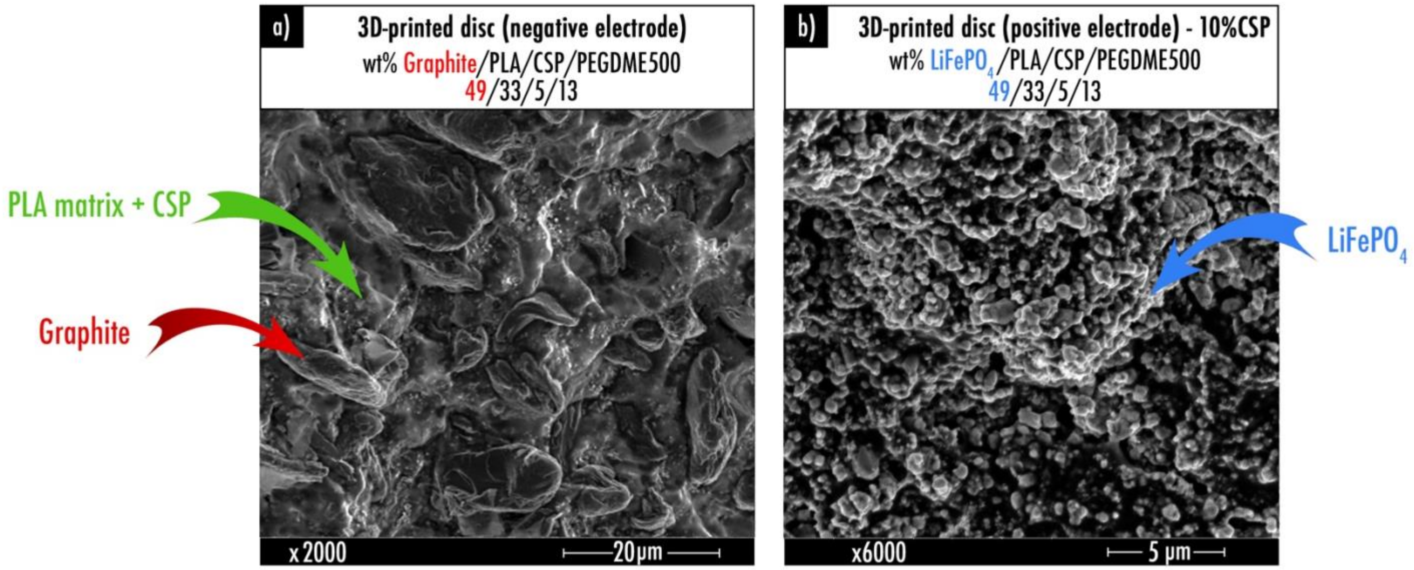

Figure S3. SEM images depicting the microstructure of the (a) 3D-printed disc prepared for negative electrode (cf. ref 42); (b) 10%CSP 3D-printed disc sample prepared for the positive electrode.

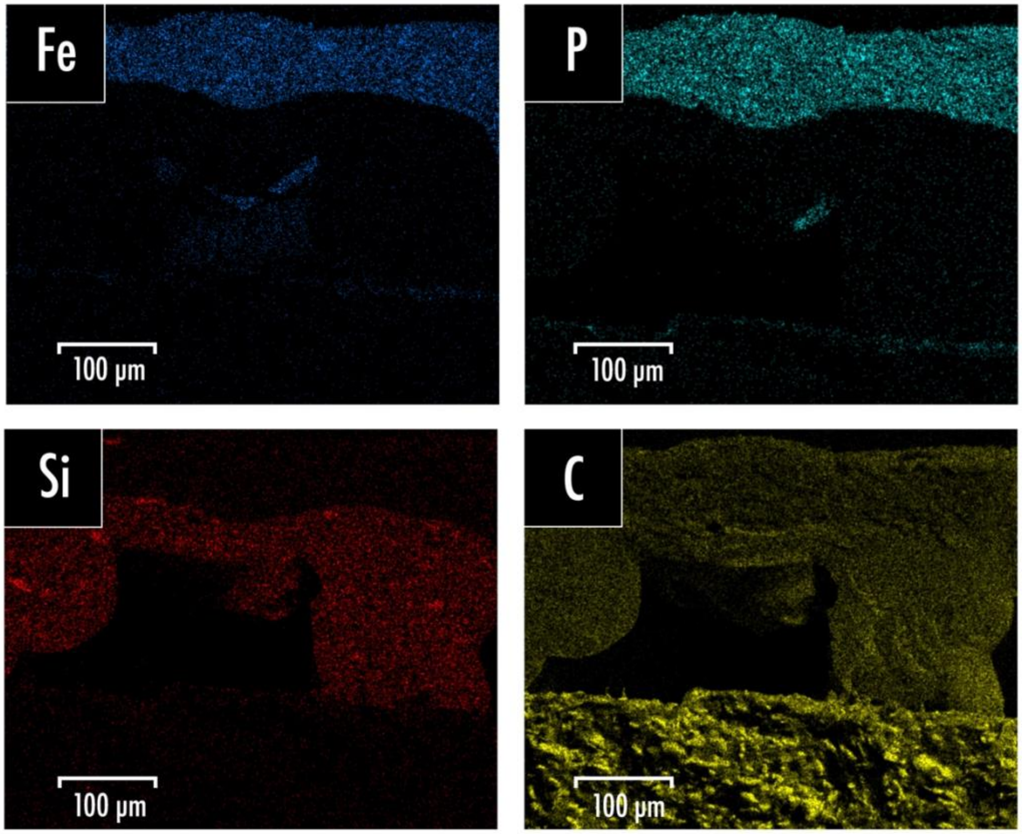

Figure S4. Element distribution map (Fe, P, Si, C) was obtained by Energy Dispersive X-ray Spectroscopy (EDS).
